# Conserved Structural Motifs of Two Distant IAV Subtypes in Genomic Segment 5 RNA

**DOI:** 10.3390/v13030525

**Published:** 2021-03-22

**Authors:** Paula Michalak, Julita Piasecka, Barbara Szutkowska, Ryszard Kierzek, Ewa Biala, Walter N. Moss, Elzbieta Kierzek

**Affiliations:** 1 Institute of Bioorganic Chemistry, Polish Academy of Sciences, Noskowskiego 12/14, 61-704 Poznan, Poland; paulineblackink@gmail.com (P.M.); julitak@ibch.poznan.pl (J.P.); barbaraszutkowska90@gmail.com (B.S.); rkierzek@ibch.poznan.pl (R.K.); ewabiaa@gmail.com (E.B.); 2 Roy J. Carver Department of Biophysics, Biochemistry and Molecular Biology, Iowa State University, Ames, IA 50011, USA; wmoss@iastate.edu

**Keywords:** RNA structure, influenza A virus, RNA conserved motifs, chemical mapping

## Abstract

The functionality of RNA is fully dependent on its structure. For the influenza A virus (IAV), there are confirmed structural motifs mediating processes which are important for the viral replication cycle, including genome assembly and viral packaging. Although the RNA of strains originating from distant IAV subtypes might fold differently, some structural motifs are conserved, and thus, are functionally important. Nowadays, NGS-based structure modeling is a source of new in vivo data helping to understand RNA biology. However, for accurate modeling of in vivo RNA structures, these high-throughput methods should be supported with other analyses facilitating data interpretation. In vitro RNA structural models complement such approaches and offer RNA structures based on experimental data obtained in a simplified environment, which are needed for proper optimization and analysis. Herein, we present the secondary structure of the influenza A virus segment 5 vRNA of A/California/04/2009 (H1N1) strain, based on experimental data from DMS chemical mapping and SHAPE using NMIA, supported by base-pairing probability calculations and bioinformatic analyses. A comparison of the available vRNA5 structures among distant IAV strains revealed that a number of motifs present in the A/California/04/2009 (H1N1) vRNA5 model are highly conserved despite sequence differences, located within previously identified packaging signals, and the formation of which in in virio conditions has been confirmed. These results support functional roles of the RNA secondary structure motifs, which may serve as candidates for universal RNA-targeting inhibitory methods.

## 1. Introduction

The family of RNA viruses is of great concern as one of the most threatening to humans. Their evolutionary volatility facilitates crossing interspecies barriers, followed by fast adaptation to a new host and efficient avoidance of its immune system [1,2]. A recently published, comprehensive study of the characteristics of pathogens with high pandemic risk showed that RNA viruses were, and perhaps will be, responsible for the most dangerous pandemics [3]. Currently, many zoonotic IAV strains have been described, which are the most dangerous within the influenza family. Notably, the avian (A/H5N1) and swine (A/H1N1) influenza strains cause severe respiratory diseases and are characterized by high human mortality rate, when compared to the mild seasonal strains [4,5,6]. The genome of IAV (13 kb) consists of eight single-stranded, negative-sense RNA segments (vRNAs) [7]. Error-prone replication systems create a mutational potential called antigenic drift [8]. Meanwhile, the segmented genome structure allows the exchange (antigenic shift) of vRNA between two co-infecting strains, and is a basis for the production of new influenza subtypes with potential to cause global pandemics [8].

The vRNA has been shown to fold into complex secondary structures and to contain motifs which play many important roles within the cell. Hence, every stage of the IAV replication cycle is RNA-structure dependent [9,10,11,12]. To date, in vitro secondary structure models based on experimental data have been proposed for full-length vRNA5, vRNA7, and vRNA8 of A/Vietnam/1203/2004 (H5N1) [13,14,15]. In virio selective 2′-hydroxyl acylation analyzed by primer extension and mutational profiling (SHAPE-MaP) of the whole influenza genome of A/WSN/1933 (H1N1) strain has also been published [16]. Recently, in virio studies using a number of high throughput methods provided new information on the structures and interactions of A/Puerto Rico/8/34 (H1N1) and A/WSN/1933 (H1N1) [17,18]. In vitro and in virio studies complement each other, as the mapping results in in virio conditions may be affected by multiple factors (e.g., interactions with other RNAs and proteins) which do not occur in in vitro analyses, making the latter easier to identify and interpret [19]. Since all these factors also influence secondary structure predictions, it is essential to thoroughly analyze and couple data from different experimental approaches to increase accuracy. It has been proposed that the secondary structure motifs take part in vRNA-vRNA interactions which are important for virion assembly and which affect reassortment outcome [20,21,22,23,24,25]. Although the evolution of new strains is in constant progress, many similarities between strains exist that are necessary for their functionality and genome compatibility [21,23,26]. Secondary structure motifs which are conserved among distinct strains are important for the influenza virus to maintain its integrity and replication potential. The importance of the RNA structure was confirmed by mutagenesis and reverse genetics [17,21,22,23,27,28]. The identification of these motifs facilitates our understanding of the replication cycle and viral biology, and allows us to identify potential new targets of antiviral therapy. The recent SARS-CoV-2 pandemic showed that more universal antiviral therapies are a high priority, as developing new vaccines is very time-consuming, labor-extensive and the production is still very low-throughput [29]. Moreover, therapies depending on protein might quickly become useless due to the potential of RNA viruses to evade such drugs through evolved resistance [30,31,32,33]. Our previous studies concerning RNA structure contributed to the development of anti-influenza inhibitors directly targeting the RNA structural motifs that showed high antiviral potential when tested in cells [15,34,35,36,37].

In this study, we determined the secondary structure of the influenza A virus vRNA5 of the A/California/04/2009 (H1N1) strain. The structure prediction was based on experimental data from dimethyl sulfate (DMS) chemical mapping and selective 2′-hydroxyl acylation analyzed by primer extension (SHAPE) using N-methylisatoic anhydride (NMIA), supported by base-pairing probability calculations and comparative sequence analyses. This revealed that a number of motifs present in the proposed secondary structure model are highly conserved among distinct influenza strains and present also in in virio conditions. Furthermore, the distribution of these motifs in the regions known to take part in the genome packaging suggests their role in the process. Similarities in the vRNA structure of the NP-coding segment 5 between distant and highly pathogenic IAV strains, despite sequence variability, support its function. The experimentally determined structure of influenza RNA and knowledge regarding its conservation can serve as indicators of important targets for new inhibitory approaches. Our study not only deepens our understanding of vRNA structure and its function, but may also lead to the development of universal antivirals targeting distant IAV strains that possess structural homologs of these revealed motifs.

## 2. Materials and Methods

### 2.1. Oligonucleotide Synthesis and Labeling

All the oligonucleotides used in the study were synthesized on a MerMade12 synthesizer, deprotected, purified via PAGE electrophoresis and fluorescently labelled as described previously [15,38]. For the reverse transcription reaction, four different fluorophores were used (FAM, ROX, TAMRA and JOE). All the oligonucleotides sequences are presented in the Supplementary Data.

### 2.2. Synthesis of DNA Template of vRNA5 A/California/04/2009

The DNA template for in vitro transcription of A/California/04/2009 vRNA5 was prepared as follows. The MDCK cells were infected with the viral stock (a gift from Prof. Luiz Martinez-Sobrido, University of Rochester) with MOI = 0.1. The infected cells were incubated for 24 h at 33 °C (5% CO_2_, 95% humidity). Then, the total RNA was isolated with Trizol reagent according to manufacturer’s protocol, followed by the reverse transcription reaction (SuperScript III, Invitrogen, Carlsbad, CA, USA) using specific primer matching 3′ end of vRNA5. During the next step, the PCR reaction (PfuPlus! polymerase, EurX, Gdansk, Poland) with the specific primers containing EcoRI (on 5′ end) and PstI (on 3′ end) was carried out. All primers are listed in Table S1 in Supplementary Materials. The PCR product was purified using a purification kit (PCR/DNA Clean-up, Eurx). The final template was cloned into pUC19 plasmid. The sequence of vRNA5 was confirmed via Sanger sequencing.

### 2.3. Transcription In Vitro

A template for the in vitro transcription was prepared via PCR reaction with primers containing T7 promoter sequence. Next, 1 µg of PCR product of vRNA5 was transcribed with Ampliscribe T7 Transcription Kit (Epicentre, Madison, WI, USA) according to manufacturer’s protocol, followed by on-column purification (Rneasy MinElute, QIAGEN, Hilden, Germany). Before the experiment, the transcription product was checked for integrity via agarose gel electrophoresis according to RiboRuler High Range RNA Ladder (ThermoFisher, Waltham, MA, USA).

### 2.4. Chemical Mapping

First, 2 pmol of RNA was folded in 1× folding buffer (300 mM NaCl, 5 mM MgCl_2_, 50 mM HEPES pH 7.5) at 65 °C for 5 min and slowly cooled down to 37 °C before mapping. Chemical mapping was performed at 37 °C with NMIA (3.3 mM) for 40 min or DMS (4.6 mM) for 15 min, respectively. For the negative control, RNA was treated with DMSO (NMIA control) or 96% ethanol (DMS control) only. The reactions were ethanol precipitated before the next step.

### 2.5. Reverse Transcription and Primer Extension

The reverse transcription (RT) reaction was performed as described previously [15]. For the reaction, a set of eight primers was used to read whole vRNA5 (Supplementary Materials Table S2), as a few premature reverse-transcription stops were observed. The reactions were performed separately in at least three independent replicates in vitro. For the reaction (+), FAM-labelled primer was used and JOE-labelled primer for the control reaction (−). Two ddNTP sequencing ladders were prepared with TAMRA or ROX labelled primers as described in Michalak et al. [15]. All the reactions (reaction (+), control (−) and two ddNTP ladders) were combined and separated on single-capillary electrophoresis.

### 2.6. Data analysis

#### 2.6.1. Primer-Extension Data Analysis

The results of capillary electrophoresis (ABI files) were analyzed with ShapeFinder software [15,39]. The data were normalized using a 2–8% normalization method. Briefly, the reactivity values were sorted decreasing from the highest, and 2% of them were excluded from the latter calculation, while 8% of the remaining values were used for average reactivity calculation. For normalization, all reactivities were divided by the average value. Reactivities ≥0.700 were considered strong, 0.700–0.500 medium and <0.500 weak. Nucleotides with no reactivity data were indicated as −999. Independent primer-extension reactions were compared to calculate the average reactivities of every nucleotide.

#### 2.6.2. Secondary Structure Prediction and Base-Pairing Probability Calculation

The secondary structure of vRNA5 was predicted using the RNAStructure 5.8.1 software [40]. Three approaches were used for the determination of vRNA5 secondary structure. At first, experimental data from vRNA5 chemical mapping were used: constraints from SHAPE as pseudo-energies and DMS constraints including strong reactivities together with conserved panhandle base-pairs. Secondly, experimental data from chemical mapping of A/California/04/2009 (H1N1) and A/Vietnam/1203/2004 (H5N1) (from Michalak et al. [15]) were used simultaneously to determine the vRNA5 structure with Dynalign algorithm [41,42]. Finally, pseudo-energy constraints from SHAPE-mapping as well strong DMS mapping constraints were implemented to the RNAStructure 5.8.1 program. For more accurate structure prediction, the consensus base-pairs with 100% probability, as calculated previously [15], were also constrained in the prediction. Average SHAPE data constraints, as well as DMS mapping results and base-pairs used in the prediction, are provided in Supplementary Materials (Tables S3 and S4). Base-pairing probabilities of vRNA5 structure model were calculated according to experimental SHAPE pseudo-energy constraints and DMS constraints (used for folding) via partition function (RNA Structure 5.8.1). The generated dot plot file was visualized in IGV (version 2.8.9) [43].

#### 2.6.3. Conservation of Canonical Base-Pairing Calculation

For canonical base-pairing calculation, 39,364 sequences of segment 5 coding RNA from the NCBI Influenza Virus Database were used [44]. Sequences were converted to reverse complement and aligned via MAFFT program (FFT-NS-1) [45]. The vRNA5 structure model was mapped to the alignments before base-pairing calculation, giving the percentage conservation of canonical base-pairing (GC, AU and GU, respectively). The number of inconsistent, or potentially noncanonical, pairs was counted, followed by percentage calculation of conservation. The results were manually checked to identify potential structure-preserving changes by recognizing consistent or compensatory mutations (Supplementary Materials Table S4).

## 3. Results and Discussion

### 3.1. Results of Chemical Mapping

**Figure 1:**
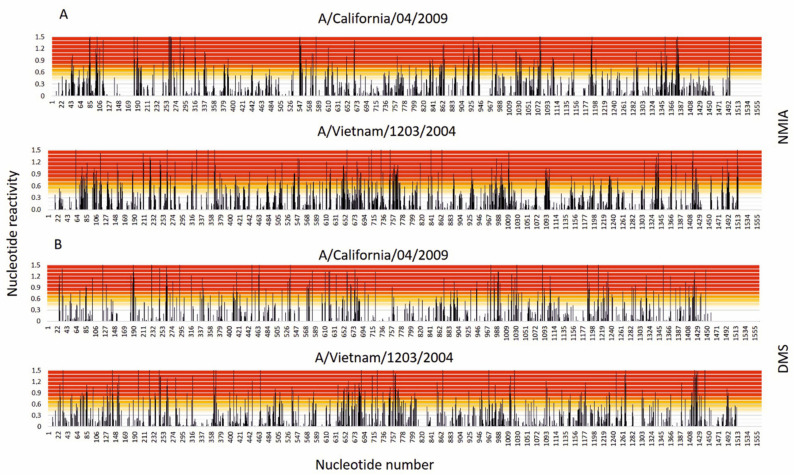
Nucleotide reactivities across vRNA5 in two subtypes of IAV strains (**A**,**B**)—A/California/04/2009 (H1N1) (California) and A/Vietnam/1203/2004 (H5N1) (Vietnam). The colors indicate the reactivity strength–red indicates strong reactivities ≥0.7, medium reactivities are marked with yellow 0.7–0.5, while low or no reactivity <0.5 is marked with white.

Chemical mapping experiments with DMS and NMIA enabled us to identify reactive nucleotides in vRNA5. The results showed that 125 nucleotides were strongly modified with DMS, meaning that 16.5% of all adenosines and cytidines in vRNA5 were single stranded and accessible. Distribution of nucleotides modified with NMIA was even for the most part of vRNA5. Only a few regions showed stronger reactivity, manifesting in short stretches of several modified nucleotides in a row: 182–198 nt, 259–264 nt, 547–553 nt, 854–867 nt, 1071–1081 nt, 1377–1383 nt. Two rather long RNA regions were identified in which no modification was observed: 131–181, 1235–1288 nt. To date, some structural data on vRNA5 originating from different influenza A strains have been published. One of the reports concerns the entire vRNA5 secondary structure of A/Vietnam/1203/2004 (H5N1) [15]. The structure was determined in vitro on the basis of chemical mapping experiments supported by isoenergetic microarray mapping, free energy minimization and bioinformatic analysis. Sequence identity between these two strains is 83.3%. The distribution of the most and least reactive regions in vRNA5 from A/Vietnam/1203/2004 (H5N1) strain is comparable to vRNA5 from A/California/04/2009 (H1N1) strain (Figure 1).

### 3.2. Global Folding of vRNA5 A/California/04/2009

The prediction of the RNA secondary structure of long molecules is challenging. The implementation of experimental data obtained for vRNA5 (1565 nt) resulted in the prediction of many structures with subtle free energy differences, with no indication of which among them was most likely to occur in native conditions. Hence, we performed several approaches for structure folding. Finally, for the conclusive structure prediction, we decided to additionally implement data from a bioinformatic analysis of the base-pairing conservation in type A influenza. These were published already by Michalak et al [15]. In our view, experimental data coupled with a bioinformatic structure conservation/homology analysis resulted in a more accurate secondary structure prediction, as proved in previous reports [46,47,48].

To determine the secondary structure of vRNA5, firstly, the experimental SHAPE data as constraints and strong DMS mapping (0.7≥) along with base-pairing of conserved promoter (panhandle) region were implemented in the RNAStructure 5.8.1 program. The obtained structure is presented in Figure S1. Next, to determine the lowest free energy vRNA5 structure according to experimental data from independent chemical mapping of vRNA5 from distant strains, we performed an additional analysis with the Dynalign algorithm. In detail, Dynalign is able to predict common secondary structures for two RNAs on the basis of the sequence alignment and experimental constraints coupled with calculation of free energy minimization with nearest neighbor parameters. It is worth noting that the Dynalign algorithm makes it possible to introduce chemical mapping constraints alone (DMS data), without soft constraints from SHAPE data (Materials and Methods). Globally, the structure predicted with Dynalign (Figure S2) showed domain conservation and indicated the presence of structural motifs common for both A/Vietnam/1203/2004 and A/California/04/2009 vRNA5. Interestingly, the initially predicted structure of A/California/04/2009 vRNA5 (Figure S1) was globally different, with preservation of few common structural motifs.

**Figure 2:**
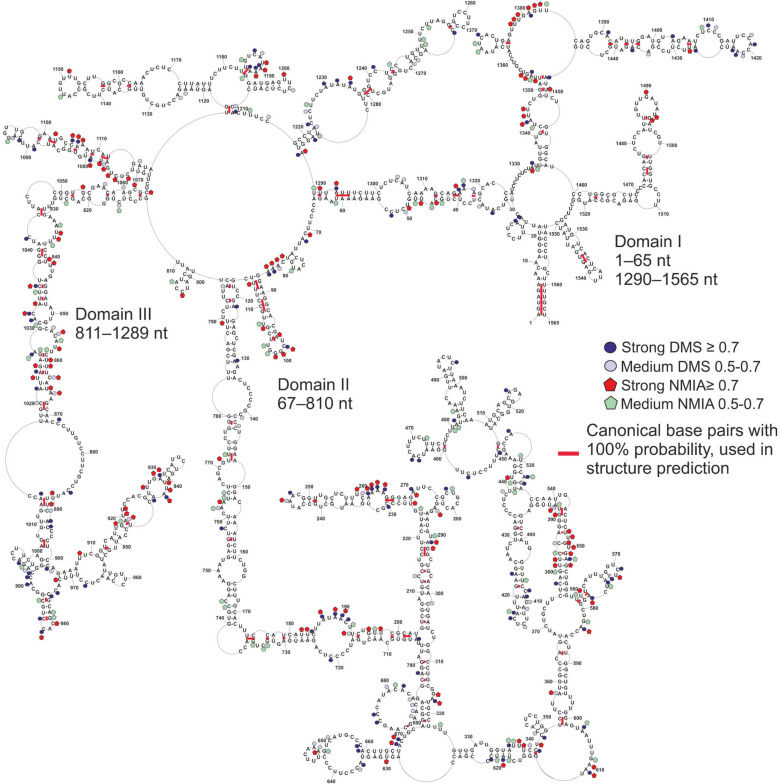
The secondary structure of vRNA5 A/California/04/2009 predicted using experimental data. Strong modifications of DMS and NMIA were marked on the structure. The canonical base-pairs with 100% of probability used in structure prediction are marked with red.

For the final structure prediction, we decided to combine the empirical data (SHAPE and DMS constraints) with base-pairing data of the conserved base-pairs from our sequence-structure bioinformatic analysis of type A influenza. Importantly, we restricted the base-pairing data to those having 100% conservation within all IAV strains [15]. The used base-pairing constraints were in agreement with experimental reactivities from chemical mapping of A/California/04/2009 vRNA5 (Figure 2).

The predicted vRNA5 secondary structure is complex and consists of three domains (Figure 2). Domain I contains the motifs between nucleotides 1–65 nt and 1290–1565 nt. Additionally, nucleotides 1–16 and 1565–1551 form a panhandle motif, recognized by viral RNA-dependent RNA-polymerase, which is conserved among all segments and IAV strains [11]. Domain II and III encompass nucleotides 67–810 and 811–1289, respectively. The secondary structure of vRNA5 is characterized by helices interrupted by bulges and internal loops, which generally correspond with modification sites identified by chemical mapping. There are 28 hairpins found in the structural model.

In comparison to the folding of A/Vietnam/1203/2004, for both strains, the organization of the domains in the secondary structure model is preserved. Sequence changes result in some of the base pairs being abolished, but new base pairs are also formed. As a consequence, the secondary structure undergoes subtle rearrangements. In general, A/California/04/2009 (H1N1) vRNA5 lacks the long helical fragments found in A/Vietnam/1203/2004 (H5N1), which consist of up to nine uninterrupted base-pairs (e.g., 40–47/1309–1316, 165–172/740–747, 327–334/622–629, 843–850/1031–1038, 1079–1087/1101–1103). Despite these differences, a number of motifs present in the secondary structure of A/Vietnam/1203/2004 (H5N1) vRNA5 are still preserved in A/California/04/2009 (H1N1). The panhandle motif and hairpins in regions 87–115 nt, 975–987 nt, 1256–1265 nt, 1363–1375 nt, 1527–1550 nt are present in both strains. Some of the hairpins differ only by the presence or lack of one base pair: 241–251 nt, 460–476 nt, 1183–1193 nt and 1483–1497 nt. Identification of common structural features is consistent with reports that RNA structure correlates with function. Some of the motifs are conserved to take part in processes which are important for the viral replication cycle. The panhandle motif, as an example of a known structure-function correlation, may also serve conserved hairpins 87–115 nt and 1483–1497 nt (99.5 and 87.2% conserved, respectively), identified in 5′ and 3′ terminal packaging signals [49]. These hairpins were predicted in previous studies and present in the secondary structure models of both strains. Beyond the terminal packaging signal, two other hairpins were predicted and identified as highly conserved in previous studies of A/Vietnam/1203/2004 (H5N1) vRNA5—975–987 nt (96.9% conserved, calculated for nucleotides 974–988 nt) and 460–476 nt (83%)—which are also present in A/California/04/2009 (H1N1) vRNA5.

### 3.3. Base-Pairing Probabilities Based on Experimental Data

**Figure 3:**
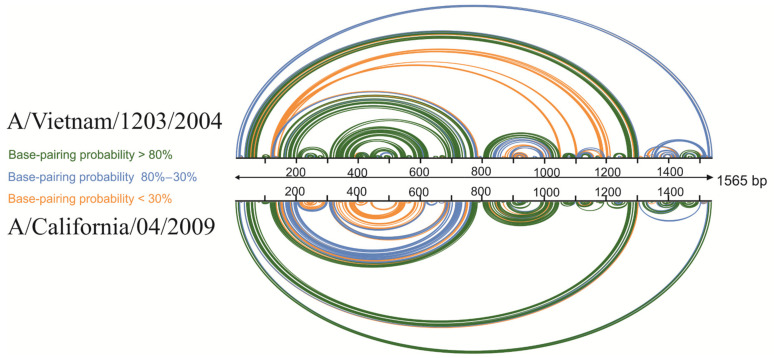
Visualization of vRNA5 global base-pairing profile and base-pairing probability based on experimental data from chemical mapping for strains A/Vietnam/1203/2004 and A/California/04/2009. The colors indicate the percentage of the base-pairing probability.

The experimental data from the previously described vRNA5 A/Vietnam/1203/2004 structure, as well as data from this study for vRNA5 structure of A/California/04/2009, were used for accurate visualization of global base-pairing profile. The comparison of probability of pairing also revealed (Figure 3) similarity in the overall global structure of the vRNA5 for both strains. In both structures, there are several regions of higher (>80%) probability of pairing including hairpin structures as well as long-distance interactions. These hairpins are in regions 87–115 nt, 1077–1112 nt, 1133–1164 nt, 1460–1522 nt, 1527–1550 nt, whereas long-distance interactions with high probability of pairing are observed in regions 37–65/1290–1318 nt and 810–839/1039–1064 nt in A/California/04/2009.

### 3.4. Conservation of Canonical Base-Pairing in vRNA5

**Figure 4:**
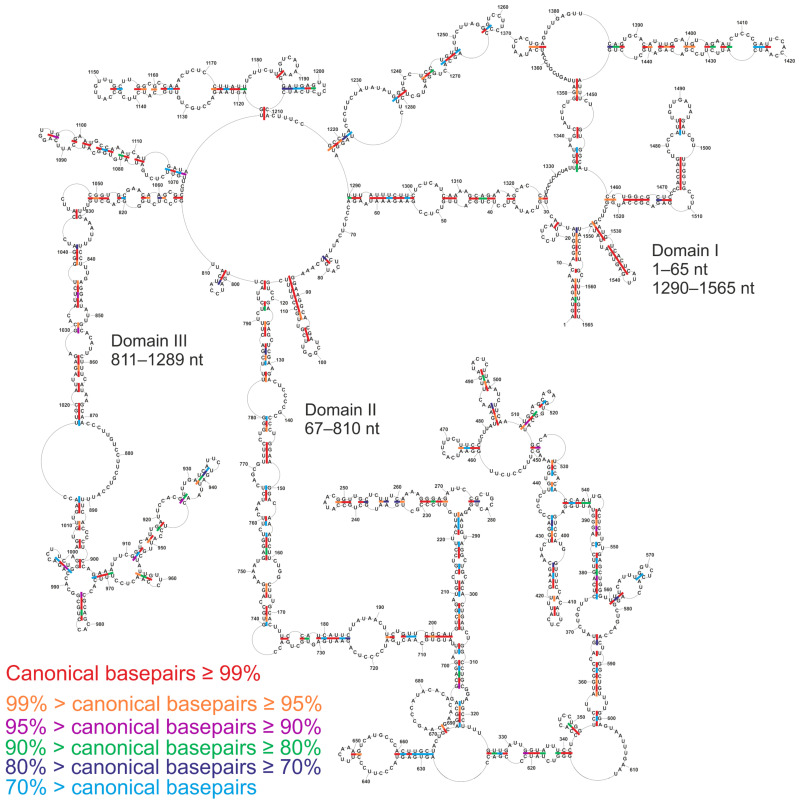
Conservation of determined vRNA5 secondary structure across influenza A viruses. Colors indicate percentage of canonical base pairing preserved among influenza A sequences for segment 5 vRNA. The analysis was done on 39,364 sequences.

The conservation of canonical base-pairing of the presented secondary structure model was calculated based on an analysis of 39,364 sequences of genomic vRNA5 within different IAV subtypes available in the database. The vRNA5 secondary structure is characterized by high base-pair conservation within IAV, on average 89.6%, confirmed by compensatory and consistent mutations (Figure 4, Supplementary Materials). There are many structural motifs and helixes with base-pairing probability higher than 95%, which are further supported by the observed structure preserving sequence variations. There are several hairpins with lower probabilities: 509–525 nt (88.8%), 911–954 nt (86.1%), 992–1002 nt (67.0%), 1194–1209 nt (77.6%) and helix 628–634/666–660 nt (64.7%). There are two hairpins with nearly 100% conservation: 1527–1550 nt and 87–115 nt. Two helixes, spanning regions 892–901/1003–1013 nt and 859–870/1027–1018 nt, are conserved within different IAV subtypes.

### 3.5. Secondary Structure of vRNA5 and Antisense Oligonucleotides (ASOs) Inhibitory Potential

In our previous study, we tested the inhibitory potential of ASOs against vRNA5 A/California/04/2009 (H1N1) [15]. At the time of publication, in the absence of experimental mapping data for this particular strain, the target regions were selected on the basis of structure predicted for vRNA5 A/Vietnam/1203/2004 (H5N1). The assumption was made that secondary structure motifs important for viral replication are conserved among strains. This conclusion was supported by a structure conservation analysis, using available influenza A sequences, showing that the proposed secondary structure of vRNA5 is 87% conserved. Potential compensatory and consistent mutations supporting predicted base pairing were found in the sequences. The current, experimentally informed, secondary structure model of vRNA5 A/California/04/2009 (H1N1) allowed us to confront the obtained results. The most effective tested antisense oligonucleotide 883–11L showed 88% replication inhibition of A/California/04/2009 (H1N1), according to the IFA assay. Interestingly, 883–11L was targeting a fully single-stranded RNA region preserved in both strains, upon which the secondary structure was modeled. The target sequence for another potent oligonucleotide (64% inhibition)—474–21M—was overlapping conserved hairpin 460–476 nt. Also, conserved hairpin 1256–1265 nt was a target for oligonucleotides 1253–13M and 1253–13L, causing 48% and 43% reduction in virus titer, respectively. These motifs were confirmed to exist in both strains. The last of the effective oligonucleotides—79–18GP—targeted the 5′ terminal packaging region, adjacent to the conserved hairpin 87–115 nt, with a high proportion of unpaired nucleotides in predicted vRNA5 A/California/04/2009 (H1N1) secondary structure. Together, these data provide structural foundation for the inhibition of the influenza virus and confirm previous assumptions. Efficient targets are conserved and accessible. The results are consistent with the expected functional role of conserved motifs, which may be targeted by antisense oligonucleotides causing a reduction of viral titer.

### 3.6. Mirror Structures in vRNA5 within Distant IAV Strains

A previous report on the vRNA5 A/Vietnam/1203/2004 (H5N1) secondary structure also confirmed the presence of previously predicted “mirror structures” in segment (−) and (+) strand [23]. Recently, a secondary structure model was also published for (+)RNA5 A/California/04/2009 (H1N1), enabling additional comparisons to be made concerning this particular strain. Current data show that two of the predicted motifs are present in both (+) and (−)RNA5 of this strain, as it was in A/Vietnam/1203/2004 (H5N1). One is a hairpin 1527–1550 nt in the (−) strand that corresponds to the 16–39 in (+) strand, which is conserved across influenza A strains and likely plays a role in the packaging of vRNA. The second is a highly conserved (96.9%) hairpin 975–987 nt in (−) and 579–591 nt (+) strand. Other proposed mirror structures include hairpins 1462–1476 nt and 626–643 nt, while pseudoknot/hairpin 36–90 nt which do not appear in the A/California/04/2009 (H1N1) vRNA5 secondary structure model, as also observed for A/Vietnam/1203/2004 (H5N1). However, there are some additional mirror structures in segment 5 A/California/04/2009 (H1N1) which were also present in A/Vietnam/1203/2004 (H5N1). In (−) and (+) strands, there are the following corresponding motifs: 1341–1454 nt and 109–225 nt, 89–113 nt and 1453–1477 nt, 460–476 nt and 1090–1106 nt, 1243–1273 nt and 293–323 nt, 1472–1506 nt and 60–94 nt, respectively. Mirror structures characteristic for A/California/04/2009 (H1N1) strain were also found: 1194–1209 nt and 357–372 nt, 409–420 nt and 1146–1157 nt in (−) and (+) strand, respectively. The functions of these motifs still remain to be elucidated. The mirror structures are presented in Figure S3.

### 3.7. NP-Binding Profile In Vivo Influences ASOs Inhibitory Potential

Previous studies concerning the A/California/04/2009 (H1N1) strain revealed an irregular NP-vRNA5 binding profile in vRNP complexes [50]. This feature allows functional secondary structure motifs to form predominantly in the RNA regions with reduced NP binding. Besides the panhandle motif, the following sites with poor NP association were identified in vRNA5: 330–490 nt, 600–640 nt, 750–950 nt and 1140–1400 nt. Despite some differences in NP-association observed between A/California/04/2009 and A/WSN/1933 strains in the report, the abovementioned regions show similar reduced NP binding. These regions were targets for four out of the five antisense oligonucleotides which were found to be most potent in the A/California/04/2009 (H1N1) inhibition (883–11L, 474–21M, 1253–13L, 1253–13M) [15]. Other tested oligonucleotides were targeting regions presenting medium association with NP; among them was also 79–18GP, which inhibited influenza virus replication by more than 40%. Therefore, it is possible that uneven the binding of NP in these regions leaves specific sites accessible for oligonucleotides.

### 3.8. The Structure of the vRNP5 Complex Revealed Interacting Regions

The exposed RNA fragments in the vRNP may have a role in RNA interactions at intramolecular (e.g., in formation of higher order structures) and intermolecular levels with other RNAs (e.g., during viral progeny packaging). This is also supported by analyses indicating that the RNA within the identified interaction loci is highly structured, which emphasizes the functional role of structural motifs [16]. Moreover, the interactions are energetically highly favorable. At the same time, experimental data show that RNA interactions present some degree of plasticity. A comparison of the interaction loci between A/WSN/1933 and A/Puerto Rico/8/34 (H1N1) strains demonstrated a common core of the network, while investigation of the distantly related A/Udorn/72 (H3N2) strain revealed more differences. However, the distribution of the most prevalent interactions between segments, even for one particular strain, may vary. To date, according to experiments carried out on the abovementioned strains, vRNA5 was reported to interact with vRNA1, vRNA2, vRNA3, vRNA4, vRNA6 and vRNA7 [16]. However, according to a recently published study, vRNA5 is characterized by the greatest number of interactions among other segments, and RNA-RNA contacts are formed with each of the vRNAs [17]. The most predominant interaction site was detected in the vRNA5 central region, adjacent to a strong NP-binding region. The presence of NP does not preclude RNA-RNA contacts, since interactions were also identified in the NP-abundant regions. In general, multiple interactions were associated with vRNA5 regions 57–81, 82–106, 482–506, 657–687, 682–706, 907–931, 957–981, 1432–1456, and 1482–1506. Interestingly, some of these regions are known to encompass previously identified, highly conserved hairpins, recurrent in different strains (87–115, 975–987, 1483–1497). Mutagenesis of region 656–705 using synonymous codons caused the loss of the hotspot and interaction rearrangements. As a consequence, new prevalent loci were formed in vRNA3, 4, and 6 to create thermodynamically favorable duplexes. It is expected that sites of interactions contain functional RNA structural motifs which take part in the packaging of progeny virions. As part of the interaction, loci are common, regardless of strain; also, secondary structure motifs may be preserved. It was also shown that prominent interaction loci can determine the reassortment process due to their influence on segment cosegregation [16]. The interactions present in the reassorted viruses overlap with those observed in parental strains. This observation led to the conclusion that RNA structure is an important factor affecting the ability of segments from different viral strains to undergo reassortment and produce new strains.

### 3.9. The Secondary Structure of vRNA5 within Different IAV Strains

Recently, an in virio and ex virio SHAPE-MaP analysis of the influenza A virus A/WSN/1933 (H1N1) strain genome structure was published [16]. The secondary structures presented in the abovementioned paper lacked long-distance base-pairing. RNA folding predictions were set up to predict local constrained RNA structures at a maximum base pairing distance limited to 150 nucleotides. Such restrictions were not considered in the modeling of vRNA5 secondary structure presented herein. Different approaches to RNA structure analysis obviously affect the obtained results and limit the scope of comparison. However, among the vRNA5 local motifs, there are some which are consistent with the herein determined secondary structure of A/California/04/2009 (H1N1) vRNA5 (described in Table 1). Hairpins 460–476 nt, 993–1001 nt, 1194–1209 nt, 1527–1550 nt are preserved in virio with base-pairing probability of more than 80%. Additionally, a pseudoknot was also predicted in region 79–154 nt. This pseudoknot was also proposed by other authors [18,22,23]. However, the base-pairing probability in this region does not exclude the formation of a hairpin at 91–111 nt, as determined in our structural model, which may play a role in packaging. Moreover, there are also hairpins predicted with lower base-pairing probability in stem regions 406–422 nt, 931–941 nt, 976–986 nt, 1363–1375 nt, 1484–1496 nt. From these 10 motifs found to be preserved in the in virio investigated structure of vRNA5, eight were additionally confirmed by chemical mapping in ex virio conditions. Among structures predicted in both analyses (in virio/ex virio), there were differences in base-paring probabilities for certain motifs. Hairpins 976–986 and 1527–1550 were also determined in previous studies [23]. A region of low reactivity spanning nucleotides 87–130 in vRNA5 was also detected and confirmed in a recent comprehensive study of structure and interactions of A/Puerto Rico/8/34 (H1N1) strain [18]. The consistency of the results obtained for naked vRNA, vRNA in vRNP complex, and within virion, suggested secondary structure conservation across these forms. This region was identified as being engaged in the intersegment interactions with vRNA3, 6, and 8, supporting the functional role of RNA structural motifs. Mutations designed to disrupt the RNA structure within this region caused impaired propagation of the virus, replication and packaging of vRNA3, segment bundling, and rearrangement of vRNA-vRNA interactions.

Most of the abovementioned common motifs are located in regions known to be low NP-binding or close to 5′ and 3′ ends of the segment. The existence of the other two motifs, covering regions 976–986 nt and 993–1001 nt, support the idea that intermediate NP-binding does not exclude the formation of local secondary structures, which may be targeted by antisense oligonucleotides. Among the above listed motifs, hairpins 91–111 nt, 406–422 nt, 460–476 nt, 976–986 nt, 1363–137 nt5, 1484–1496 nt and 1527–1550 nt were previously determined also in A/Vietnam/1203/2004 (H5N1) vRNA5 in vitro secondary structure. Hairpins 460–476 nt and 976–986 nt were characterized as highly conserved, while conserved hairpins 91–111 nt and 1484–1496 nt were postulated to have a role in virion packaging. The first of them (hairpin 460–476) was mentioned previously as a potent target for antisense oligonucleotide 474–21M. Additionally, chemical mapping of three strains (A/WSN/1933 (H1N1), A/Puerto Rico/8/34 (H1N1), A/Udorn/72 (H3N2)) showed that segments with high sequence identity preserve similar RNA conformation [16]. Together, these results indicate that viral genomic RNA, regardless of the strain, shares structural features that have an important impact on its function and viral replication cycle.

Current reports on the RNA structure of the influenza virus show a spectrum of unique and common features. To some extent, discrepancies can arise from methods used in the experiments and constraints applied to structure modeling (e.g., maximum base-pairing distance of nucleotides). There are also experimental conditions which affect the results. In vitro, in vivo and other structure mapping methods have both advantages and limitations. This aspect can cause difficulties in the interpretation of data coming from many sources. The overall organization of RNA may also differ among strains. Searching for similarities and patterns among the structures may be helpful. Stable, conserved RNA structures are important for the viral replication cycle and may guide many processes which are crucial for the virus, such as packaging and reassortment. Therefore, investigation of the structural motifs of influenza RNA will improve our understanding of viral infection and evolution, and support the development of new therapeutic approaches against influenza targeting RNA.

## Figures and Tables

**Table 1:** Secondary structure motifs predicted in the in vitro mapping experiments of A/California/04/2009 (H1N1) and preserved according to in virio and ex virio mapping of A/WSN/1933 (H1N1).

Predicted Motif Nucleotide Region (nt)	Base-Pairing Probability According to Dadonaite, et al. [16]	
	In Virio	Ex Virio
91–111	<10%	none
406–422	30–80%	>80%
460–476	>80%	>80%
931–941	30–80%	10–30%
976–986	30–80%	30–80%
993–1001	>80%	<10%
1194–1209	>80%	30–80%
1363–1375	30–80%	10–30%
1484–1496	10–30%	none
1527–1550	>80%	>80%

## Data Availability

The data presented in this study are available in the article and Supplementary Materials.

## Supplementary Materials

The following are available online at https://www.mdpi.com/1999-4915/13/3/525/s1, Figure S1: The secondary structure of vRNA5 A/California/04/2009 predicted in RNAStructure 5.8.1 program by introducing experimental data from SHAPE, DMS chemical mapping, and panhandle conserved base-pairs., Figure S2. The secondary structure of vRNA5 A/California/04/2009 predicted by Dynalign algorithm in RNAStructure 5.8.1 program, Figure S3. Mirror structures present in segment 5 A/California/04/2009 (H1N1) RNA (−) and (+) strand, respectively, Table S1: DNA primers used for construction of vRNA5 DNA template in pUC19, Table S2: DNA primers used for reverse transcription, Table S3: vRNA5 nucleotide reactivities, Table S4: Base pairs counts for secondary structure of vRNA5.
