# Supplementary material for: Conserved Structural Motifs of Two Distant IAV Subtypes in Genomic Segment 5 RNA
Source: Viruses. 2021 Mar 22;13(3):525. doi: 10.3390/v13030525 (PMC8004953; doi:10.3390/v13030525)
Supplement: Supplementary file 1 [file viruses-13-00525-s001.zip › Supplementary_Figures.docx]

Supplementary Materials

Conserved structural motifs of two distant IAV subtypes in genomic segment 5 RNA

Paula Michalak ^1#^, Julita Piasecka ^1#^, Barbara Szutkowska ^1#^, Ryszard Kierzek ^1^, Ewa Biala ^1^, Walter N. Moss ^2^ and Elzbieta Kierzek ^1,^*

^1^ Institute of Bioorganic Chemistry, Polish Academy of Sciences, Noskowskiego 12/14, 61-704 Poznan, Poland

^2^ Roy J. Carver Department of Biophysics, Biochemistry and Molecular Biology, Iowa State University, Ames, IA 50011, United States of America

# P.M.., J.P., and B.S. contributed equally to this work

***** Correspondence: elzbieta.kierzek@ibch.poznan.pl


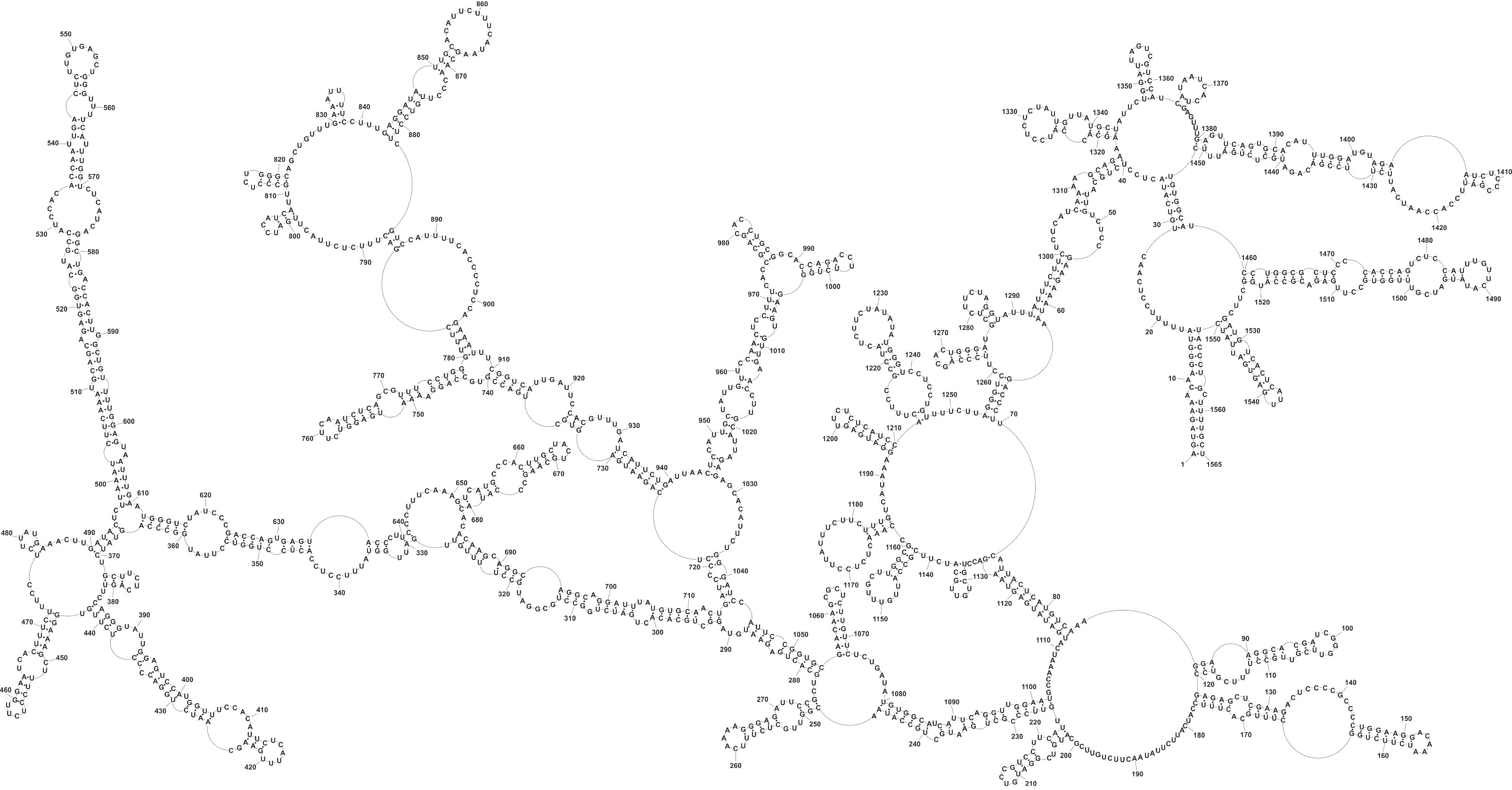


**Figure S1.** The secondary structure of vRNA5 A/California/04/2009 predicted in RNAStructure 5.8.1 program by introducing experimental data from SHAPE, DMS chemical mapping, and panhandle conserved base-pairs.


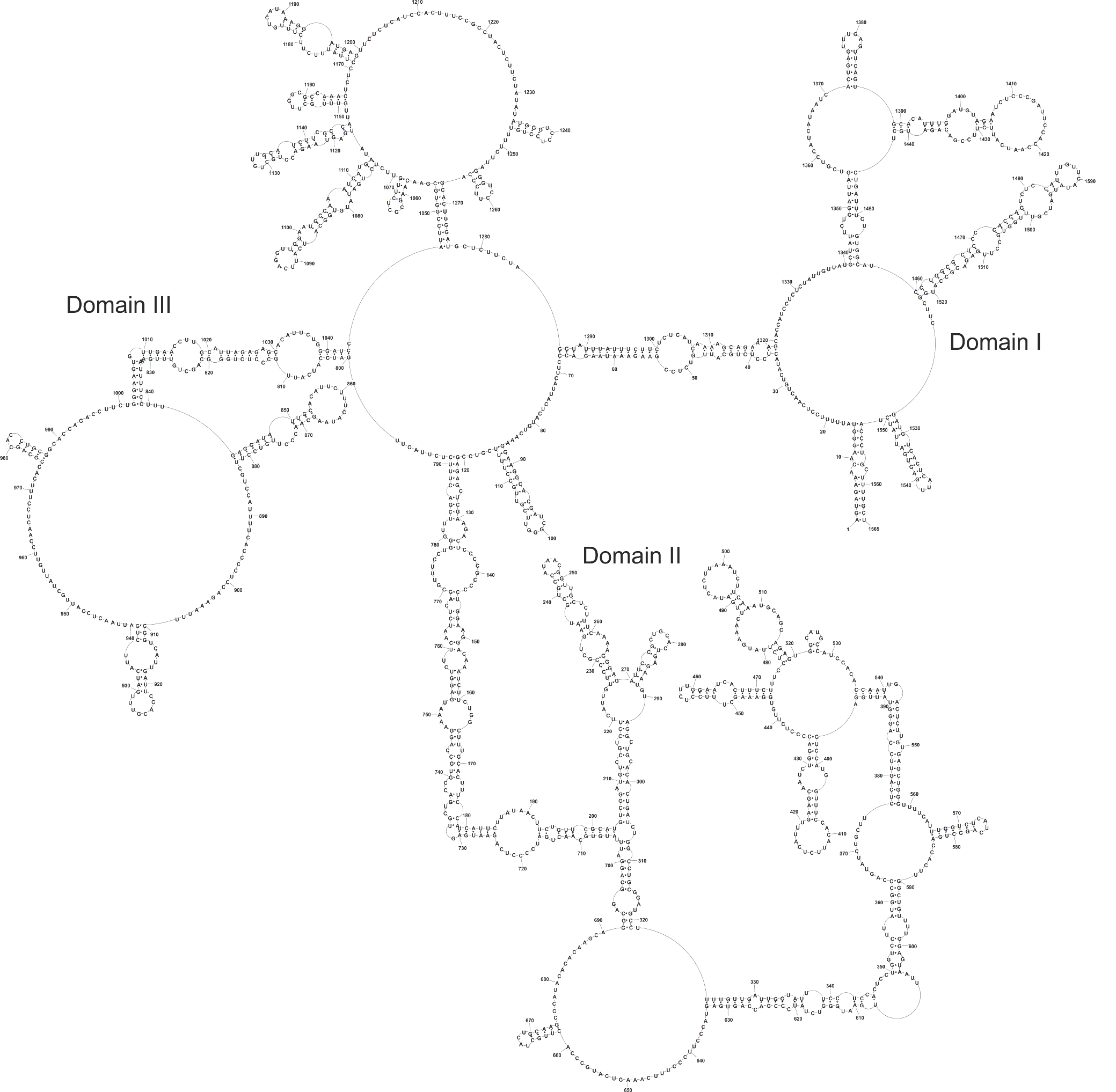


**Figure S2.** The secondary structure of vRNA5 A/California/04/2009 predicted by Dynalign algorithm in RNAStructure 5.8.1 program.


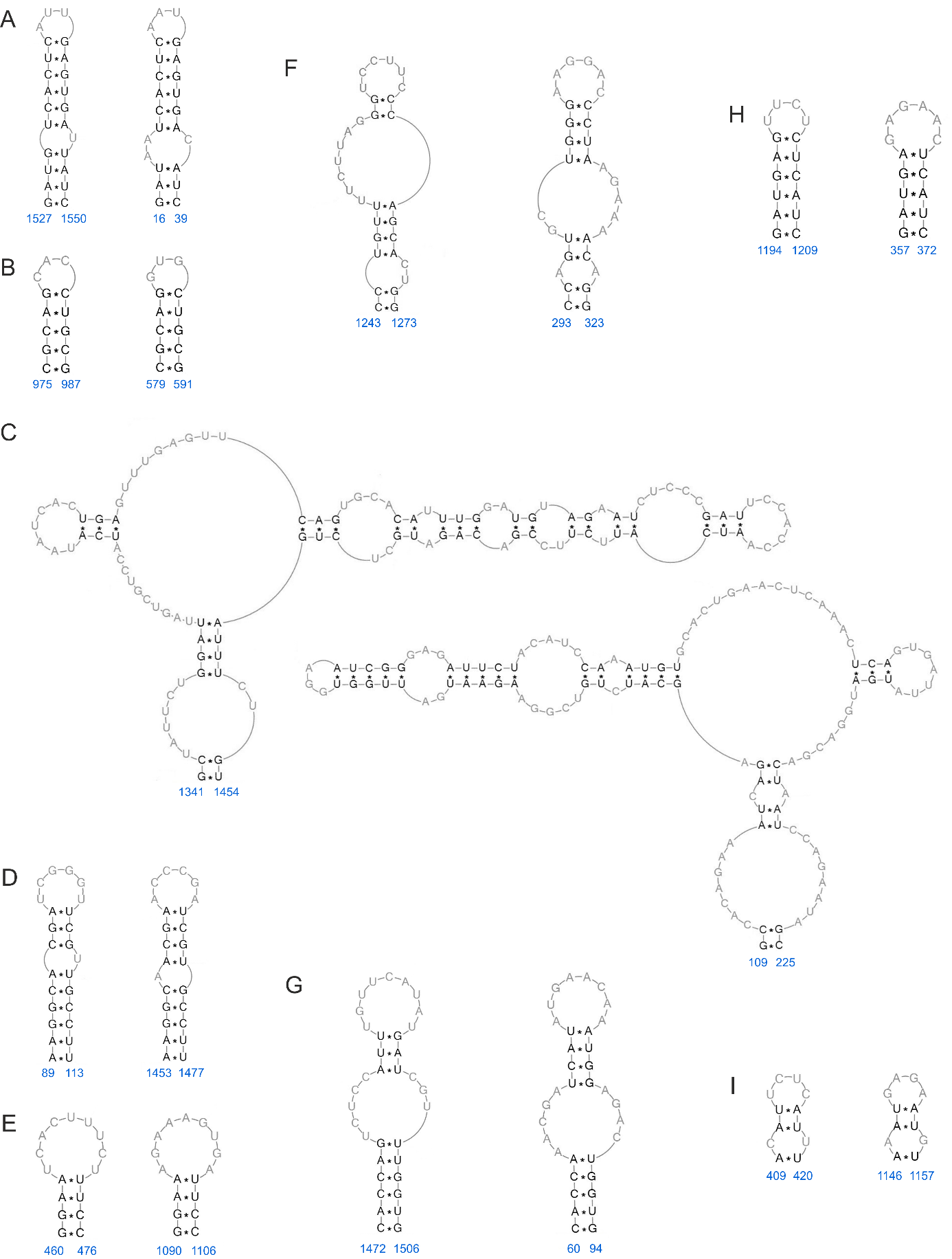


**Figure S3.** Mirror structures present in segment 5 A/California/04/2009 (H1N1) RNA (-) and (+) strand, respectively.
