# Supplementary figures and images for: Conserved Structural Motifs of Two Distant IAV Subtypes in Genomic Segment 5 RNA
Source: Viruses. 2021 Mar 22;13(3):525. doi: 10.3390/v13030525 (PMC8004953; doi:10.3390/v13030525)

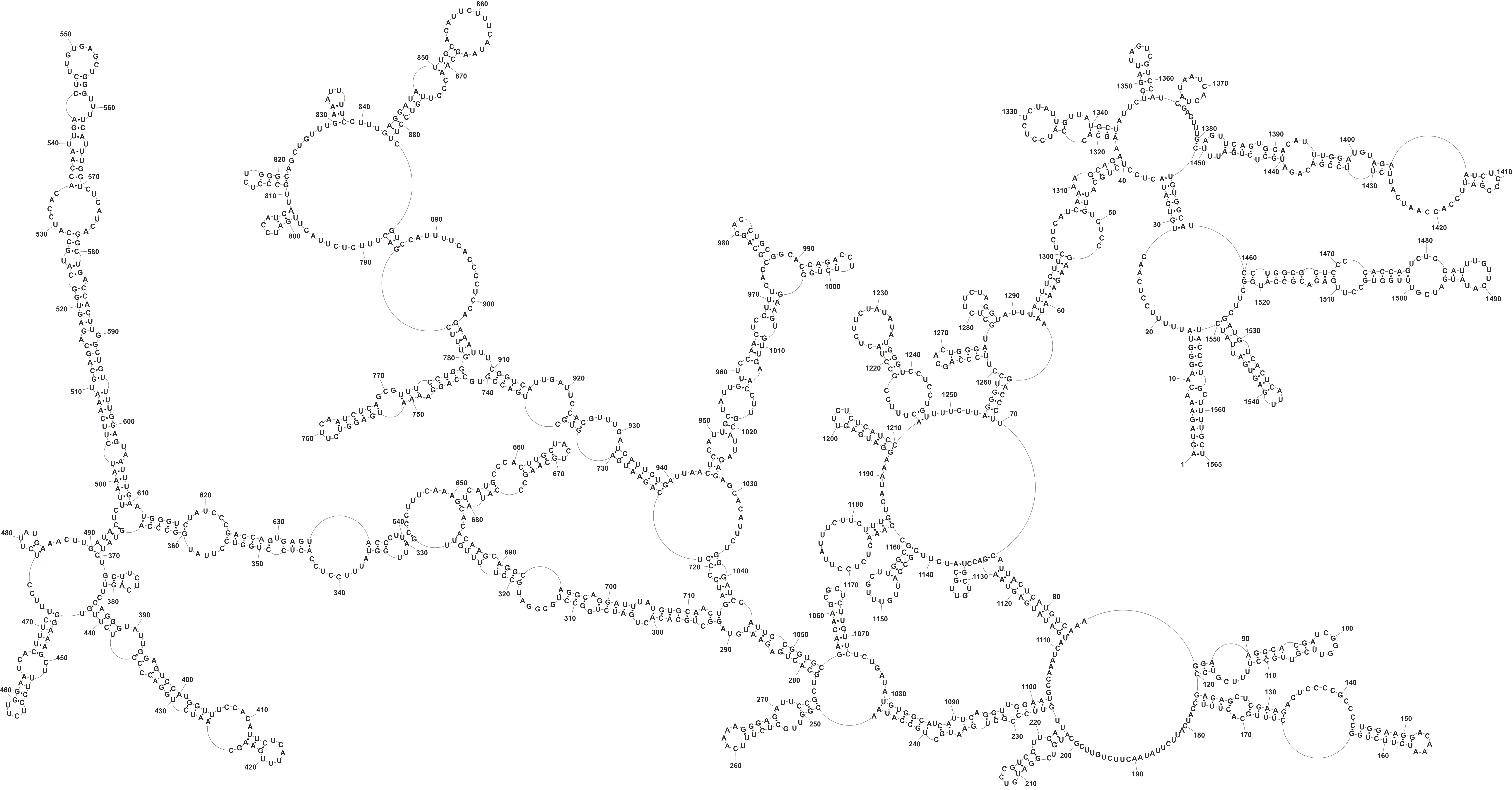

Supplement: Supplementary file 1 [file viruses-13-00525-s001.zip › Figure S1.tif]

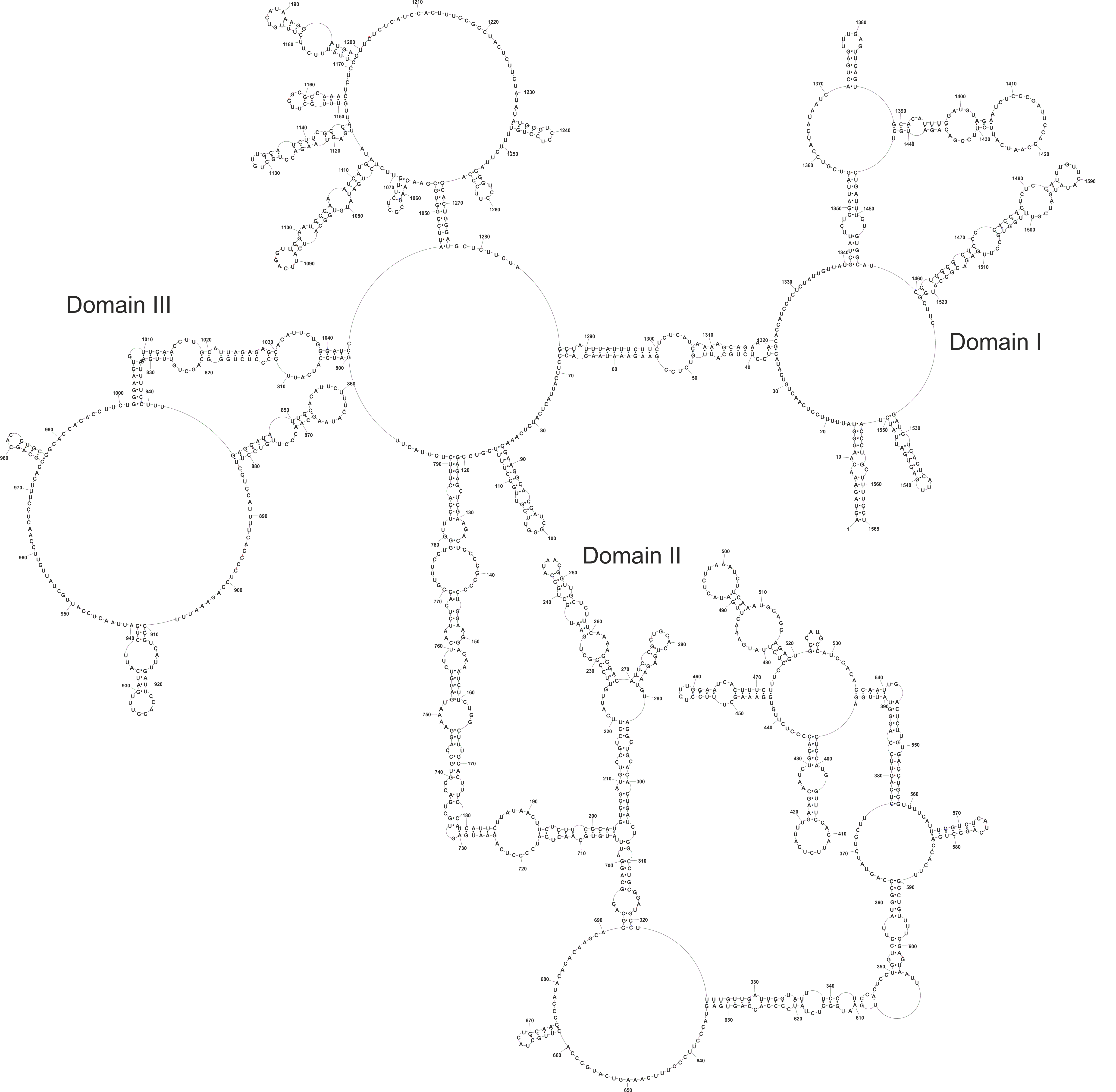

Supplement: Supplementary file 1 [file viruses-13-00525-s001.zip › Figure S2.tif]

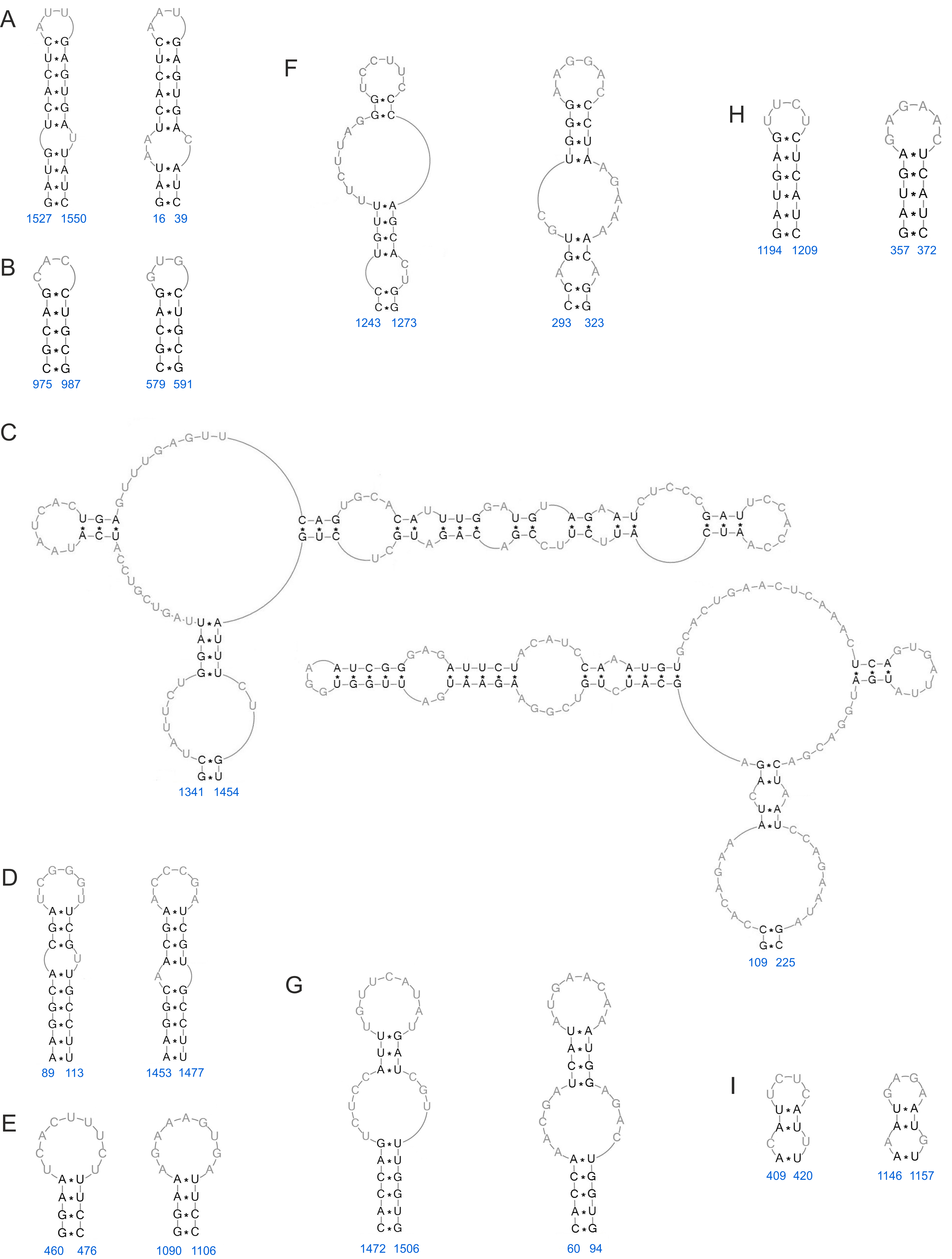

Supplement: Supplementary file 1 [file viruses-13-00525-s001.zip › Figure S3.tif]
